# Two-Step Production of Phenylpyruvic Acid from L-Phenylalanine by Growing and Resting Cells of Engineered *Escherichia coli*: Process Optimization and Kinetics Modeling

**DOI:** 10.1371/journal.pone.0166457

**Published:** 2016-11-16

**Authors:** Ying Hou, Gazi Sakir Hossain, Jianghua Li, Hyun-dong Shin, Long Liu, Guocheng Du, Jian Chen

**Affiliations:** 1 Key Laboratory of Carbohydrate Chemistry and Biotechnology, Ministry of Education, Jiangnan University, Wuxi 214122, China; 2 Synergetic Innovation of Center of Food Safety and Nutrition, Jiangnan University, Wuxi 214122, China; 3 School of Chemical and Biomolecular Engineering, Georgia Institute of Technology, Atlanta 30332, Georgia, United States of America; Virginia Polytechnic Institute and State University, UNITED STATES

## Abstract

Phenylpyruvic acid (PPA) is widely used in the pharmaceutical, food, and chemical industries. Here, a two-step bioconversion process, involving growing and resting cells, was established to produce PPA from l-phenylalanine using the engineered *Escherichia coli* constructed previously. First, the biotransformation conditions for growing cells were optimized (l-phenylalanine concentration 20.0 g·L^−1^, temperature 35°C) and a two-stage temperature control strategy (keep 20°C for 12 h and increase the temperature to 35°C until the end of biotransformation) was performed. The biotransformation conditions for resting cells were then optimized in 3-L bioreactor and the optimized conditions were as follows: agitation speed 500 rpm, aeration rate 1.5 vvm, and l-phenylalanine concentration 30 g·L^−1^. The total maximal production (mass conversion rate) reached 29.8 ± 2.1 g·L^−1^ (99.3%) and 75.1 ± 2.5 g·L^−1^ (93.9%) in the flask and 3-L bioreactor, respectively. Finally, a kinetic model was established, and it was revealed that the substrate and product inhibition were the main limiting factors for resting cell biotransformation.

## Introduction

As a multi-functional keto acid, phenylpyruvic acid (PPA) is widely used in the pharmaceutical, food, and chemical industries [1]. In the pharmaceutical industry, PPA is the starting material for the production of d-phenylalanine [2] and phenyllactic acid [3]. Currently, commercial PPA is produced by chemical processes, which causes environmental pollution [4, 5]. Enzymatic transformation is an alternative process for PPA production. d-amino acid oxidases (d-AAOs, EC 1.4.3.3) were used for the PPA production via the oxidation of either d-phenylalanine or d, l-phenylalanine, while the reaction produces toxic hydrogen peroxide [6, 7]. l-amino acid deaminases (l-AADs, EC 1.4.3.2) catalyze the oxidative deamination of l-phenylalanine to PPA without producing hydrogen peroxide and l-phenylalanine is cheaper than d-phenylalanine as substrates [8]. Therefore, l-AADs have been used as ideal enzymes for PPA biosynthesis.

In previous studies, we constructed an *Escherichia coli* whole-cell catalyst expressing an l-AAD from *Proteus mirabilis* and produced PPA in one step from l-phenylalanine with a maximal production of 3.3 ± 0.2 g·L^−1^ [9]. The metabolic pathway of PPA in *E*. *coli* was then engineered to block PPA degradation and the l-AAD was engineered, resulting in improved whole-cell catalytic performance and PPA production (10.0 ± 0.4 g/L) [10]. This one-step biotransformation showed substantial advantages such as simplified cell preparation and the capacity to recycle, but PPA titer was still not remarkably high on an industrial scale. Therefore, it is difficult to significantly improve the PPA titer by one-step biotransformation.

Two-step biotransformation involving growing and resting cells has been used in the production of many compounds by various strains, such as phenyllactic acid [3], gama-aminobutyric acid [11], and D-lactate [12] by *Lactobacillus* sp., ectoine by *Halomonas salina* [13], and betulone by *Rhodococcus rhodochrous* [14]. In brief, two-step biotransformation includes two stages, in which the product was synthesized in substrate-containing medium by growing cells and in substrate-containing buffer by resting cells, respectively.

Growing cells refer to the group of proliferating and metabolically active cells, which are provided with essential nutrients in the medium. Growing cell biotransformation is the process in which a certain amount of substrate was added to the medium and the target product was synthesized via one or several enzymatic reactions from the substrate during cell culture. The enzymatic reaction is stable due to suitable growing environment for the cells. In this study, growing cell biotransformation was applied to produce PPA via adding l-phenylalanine to the medium during the recombinant *E*. *coli* culture phase. Growing cell biotransformation is similar to the microbial fermentation, by which PPA was produced with microorganisms such as *Zygosaccharomyces rouxii*, *P*. *vulgaris*, *Morganella morganii*, and *Corynebacterium glutamicum* [15]. However, the production titer was low although many optimization strategies were used [16, 17], probably owing to the multiple steps as well as low enzymatic activities in the natural bacterial producer. Growing cell biotransformation in this study is expected to realize high production due to one-step oxidative deamination reaction and high l-AAD activity.

The resting cells are under non-growing conditions suspended in buffer solution without carbon, nitrogen, or mineral sources and are used as biocatalysts to produce many compounds [18]. Resting cell biotransformation show certain advantages over growing cell biotransformation, including simple operation steps, no need of nutrient medium, and convenient downstream product separation [19]. Modeling and simulation of resting cell biotransformation processes were widely used in better understanding the investigated process, identifying the limiting parameters, and optimizing the reaction conditions. For example, the constructed product toxicity and cometabolic competitive inhibition model by methanotrophic resting cells indicated the necessity for treatment process design [20]; the model of the biotransformation of crotonobetaine into l-(-)-carnitine by the *E*. *coli* resting cells indicated that biomass concentration strongly influenced biotransformation [21]; the kinetic study of reuterin production by *Lactobacillus reuteri* in resting cells describes simultaneously the concentrations of various parameters [22].

Therefore, in the present study, a two-step biotransformation system, growing and resting cell biotransformation, was developed to produce PPA from l-phenylalanine by metabolically engineered *E*. *coli*. The reaction conditions were optimized for facilitating cell biotransformation and a two-step temperature control strategy was developed to balance the cell growth and the two-step biotransformation. Finally, a kinetic study of resting cell biotransformation was conducted in the 3-L bioreactor.

## Materials and Methods

### Bacterial strains, chemicals, and culture conditions

The engineered *E*. *coli* BL21(DE3)(Δ*tyrB*Δ*aspC*Δ*ilvE*) harboring the recombinant plasmid pET20b-D165K/F263M/L336M were constructed in the previous study [10]. With the exception of PPA (Sigma-Aldrich, Shanghai, China), all chemical reagents were purchased from Shanghai Sangon Biological Engineering Technology and Services Co. Ltd. (Shanghai, China). The seed medium was as follows (per liter): 10 g tryptone, 5 g yeast extract, 10 g sodium chloride, and 100 mg ampicillin. The fermentation medium was as follows (per liter): 12 g tryptone, 24 g yeast extract, 4 g glycerol, 17 mmol KH_2_PO_4_, 72 mmol K_2_HPO_4_, and 100 mg ampicillin. Recombinant *E*. *coli* was inoculated into 20 mL Luria-Bertani medium and grown overnight in a rotary shaker at 37°C. A seed culture (1%, v/v) was inoculated into 50 mL fermentation medium of 500 mL Erlenmeyer flask. The standard induction conditions were as follows: pH 8, 0.04 mM IPTG, OD_600_ 0.6, and induction at 20°C for 12 h.

### PPA production by *E*. *coli* growing cells

Optimization of all variables was performed with 50 mL of reaction mixture in a 500-mL Erlenmeyer flask. The effect of initial l-phenylalanine concentrations (8.0, 12.0, 16.0, 20.0, and 25.0 g·L^-1^) on growing cell biotransformation was studied under the standard induction conditions by measuring the PPA titer every 6 h. The effect of l-phenylalanine feeding time (at the beginning of fermentation or after induction) on PPA production and cell growth was studied on the basis of the optimal l-phenylalanine concentration. The reaction was stopped by centrifugation at 10,000 × *g* for 5 min. Then, 20 μL of supernatant was withdrawn and added to 400 μL of ferric chloride (6%, w/v). The PPA amount was determined by measuring the absorbance at 640 nm [23]. l-phenylalanine was derivatized with *o*-phthaldialdehyde and measured by Agilent 1100 HPLC system (Agilent, Palo Alto, CA) equipped with a reversed-phase column (Zorbax Eclipse-AAA) at a column temperature of 30°C and UV detector at 338 nm [24]. Biomass concentration was assessed spectrophotometrically (UV-2450 PC; Shimadzu Co., Kyoto, Japan) at an optical density of 600.

Optimal biotransformation temperature was determined over a range of temperatures from 20 to 40°C under the optimal l-phenylalanine concentration and feeding time. Moreover, the effect of growing cell biotransformation on resting cell biotransformation was investigated. The growing cell biotransformation was performed under different temperatures for 24 or 28 h, after which the mixture was centrifuged at 8,000 × *g* and 4°C for 20 min and cells were washed four times with 20 mM phosphate buffer (pH 7.0). The resting cell biotransformation was started by suspending the cells in 20 mM phosphate buffer (pH 7.0) and l-phenylalanine solution (10 g·L^−1^). The control was the resting cell biotransformation without subsequent growing cell biotransformation. The resting cell biotransformation was performed under the optimal conditions (10 g·L^−1^ l-phenylalanine, 4.2 g·L^−1^ cells, pH 7.4, 40°C, 6 h) with 50 mL of reaction mixture in a 500-mL Erlenmeyer flask. Based on the optimal fermentation conditions in flask, biotransformation of l-phenylalanine to PPA was conducted using *E*. *coli* cells grown in a 3-L bioreactor (BioFlo 115, New Brunswick Scientific Co., Edison, NJ, USA) with a working volume of 1.6 L. The reaction conditions were as follows: initial pH 8.0, agitation speed 500 rpm, and aeration rate 2.0 vvm. After induction at 20°C for 12 h, 50 g·L^−1^ of l-phenylalanine was fed to the reaction system and the temperature was increased to 35°C for growing cell biotransformation.

### PPA production by *E*. *coli* resting cells in 3-L bioreactor

To optimize the agitation speed and aeration rate of resting cell biotransformation in 3-L bioreactor, the reaction conditions were 40°C, pH 8.0, 7.3 g·L^−1^ of biocatalyst (dry cell weight, w/v), and 30 g·L^−1^ of l-phenylalanine in a total volume of 1.6 L. Agitation speed between 300 and 700 rpm and aeration rate between 0.5 and 2.0 vvm were evaluated. For optimization of l-phenylalanine concentration, the reaction conditions were 40°C, pH 8.0, 500 rpm, 1.5 vvm, 7.3 g·L^−1^ of (dry cell weight, w/v) biocatalyst, and 20 to 40 g·L^−1^ of l-phenylalanine.

### Effect of dissolved oxygen, cell, substrate, and product concentrations on initial rates

To check the tendency of dissolved oxygen during resting cell biotransformation, the reaction was conducted at different l-phenylalanine concentrations (20, 30, 35, and 40 g·L^−1^) under the optimal conditions (40°C, 500 rpm, 1.5 vvm, and 7.3 g·L^−1^ of biocatalyst). The initial dissolved oxygen before adding l-phenylalanine to the reaction system was set as 100%.

The cell deactivation kinetics was conducted under different l-phenylalanine concentrations (20, 40, 60, and 80 g·L^−1^) with the optimal conditions (40°C, 500 rpm, 1.5 vvm, and 7.3 g·L^−1^ of biocatalyst). About 3 mL of reaction mixture was withdraw from the 3-L bioreactor every 15 min, after which the mixture was centrifuged at 8,000 × *g* and 4°C for 20 min and wash for four times with 20 mM phosphate buffer (pH 7.0). The cell activity was determined under the standard reaction conditions. The reaction system (pH 7.4) contained 10 g·L^−1^ l-phenylalanine and 0.6 g·L^−1^ (dry cell weight, w/v) biocatalyst in a total volume of 5 mL, and the mixture was incubated in a shaker at 40°C for 30 min. The resting cell activity was defined as the grams of PPA generated from l-phenylalanine per minute per milligram of dry cell weight.

The substrate inhibition kinetics were determined with different l-phenylalanine concentrations (from 5 to 100 g·L^−1^) under the optimal conditions. The Lineweaver-Burk plotting for the resting cell biotransformation of the mutant was performed containing different concentrations of PPA (0–20 g·L^−1^) to check the possibility of product inhibition and calculate the inhibition constant.

### Statistical analysis

All experiments were performed at least three times, and the results were expressed as the mean ± standard deviation (n = 3).

## Results and Discussion

### Optimization of growing cell biotransformation

**Fig 1:**
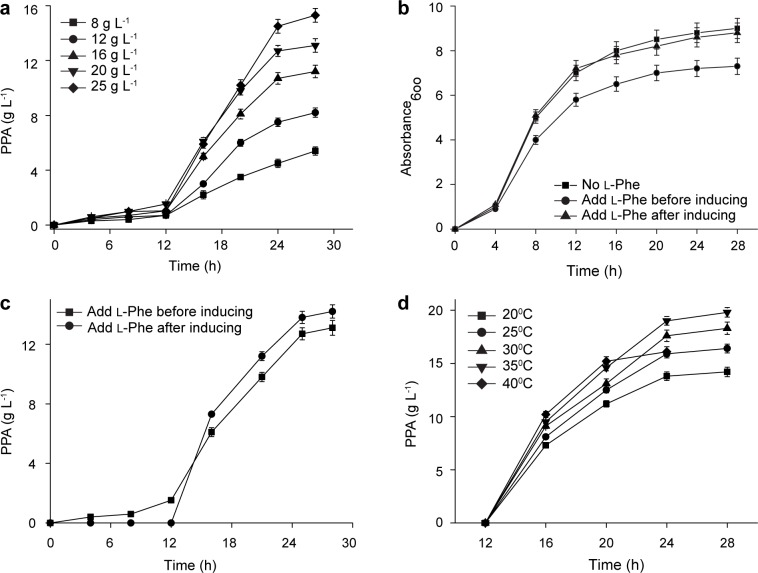
Optimization of the growing cell biotransformation in the flask. a: time profiles of PPA synthesis with different l-phenylalanine concentrations. b: time profiles of cell growth at different l-phenylalanine feeding time. c: time profiles of PPA synthesis at different l-phenylalanine feeding time. d: time profiles of PPA synthesis at different temperatures.

Our previous results show that oxidative deamination reaction is closely associated with the substrate concentration [9]. Thus, the effect of l-phenylalanine concentration on PPA production and conversion was studied (Fig 1A). The reaction velocity as well as PPA production was low at the initial stage (0 to 12 h) owing to low l-AAD expression and then increased at the later stage (12 to 24 h). PPA production increased with the initial amount of l-phenylalanine in the medium and reached the maximum level (15.3 ± 0.4 g·L^−1^) with 25 g·L^−1^ l-phenylalanine at 28 h. However, the highest conversion (65.0 ± 1.1%) was obtained with 20.0 g·L^−1^ l-phenylalanine. Further increase of l-phenylalanine concentration dramatically decreased the conversion ratio, suggesting the existence of substrate inhibition during the growing cell transformation. Therefore, the optimal l-phenylalanine concentration in the medium was 20.0 g·L^−1^. Addition of 20.0 g·L^−1^ l-phenylalanine at the beginning of the fermentation caused growth inhibition (Fig 1B). Therefore, substrate feeding was performed after induction, resulting in an increased production of 14.2 ± 0.4 g·L^−1^ (Fig 1C).

The effect of temperature on growing cell biotransformation was investigated and the time courses for PPA production at different temperatures were compared (Fig 1D). At the beginning (12 to 16 h), the reaction velocity was increased along with the temperature and reached the maximum level (2.6 g·L^−1^·h^−1^) at 40°C, which was 1.4 times that at 20°C. Next, the reaction velocity of 35°C was higher than that of 40°C. The highest PPA titers was 19.8 ± 0.4 g·L^−1^ at 35°C within 16 h, with a substrate conversion ratio of 99% and productivity of 1.2 g·L^−1^·h^−1^. Therefore, the optimal temperature (35°C) for growing cell biotransformation was lower than that of resting cell biotransformation (40°C) [9], probably because of the negative effect of high temperature on cell growth.

High temperature was unfavorable for l-AAD expression and cell growth, while a relatively high temperature was favorable for growing cell biotransformation. Meanwhile, higher temperatures in growing cell biotransformation resulted in decreased PPA production in resting cell biotransformation due to cell damage (data not shown), which is unfavorable for the total production of this two-step biotransformation. Since these three processes (l-AAD expression and cell growth, growing cell biotransformation, and resting cell biotransformation) are closely related, the temperature for growing cell biotransformation was optimized based on a comprehensive consideration of these three processes. Finally, a two-stage temperature control strategy (i.e., 20°C for 12 h, followed by addition of l-phenylalanine to the system and increased the temperature to 35°C until the end of biotransformation) was implemented. Two-stage temperature control needs less reaction time and the productivity of growing cell biotransformation was 1.4 times that of thermostatic control (20°C). Thus, this two-stage temperature control strategy is an effective strategy for improving the production.

It was reported that PPA can also be produced via microbial fermentation with many microorganisms [15]. Among these strains, *P*. *vulgaris* was the most suitable production host and the titer reached 1.3 g·L^−1^ under the optimal conditions [17]. By fed-batch fermentation, the PPA production was further improved to 3.0 g·L^−1^, which was 15.2% that of our growing cell biotransformation. Then, PPA productivity was increased to 259 mg·L^−1^·h^−1^ by conducting continuous fermentation, which was 21.6% that of our growing cell biotransformation [16]. Therefore, this growing cell biotransformation showed higher PPA production, productivity, and substrate conversion ratio than that of microbial fermentation.

### Optimization of resting cell biotransformation

**Fig 2:**
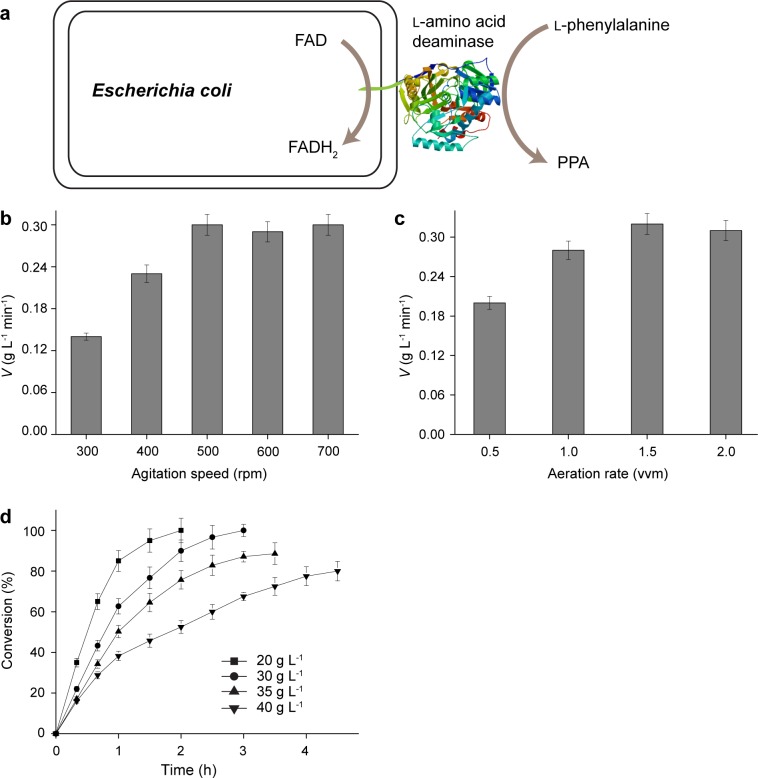
Optimization of the resting cell biotransformation in the 3-L bioreactor. a: schematic view of PPA production system by *E*. *coli* whole-cell biocatalyst. b: optimization of agitation speed. c: optimization of aeration rate. d: time profiles of conversion with different l-phenylalanine concentrations.

The schematic view of PPA production system by l-AAD-based *E*. *coli* whole-cell biocatalyst is shown in Fig 2A. The biotransformation conditions for the resting cells in the 3-L bioreactor were optimized. The oxygen requirement for biotransformation was controlled by changing the agitation speed and aeration rate. The reaction velocity increased with the increased agitation speed and reached a maximum of 0.30 ± 0.02 g·L^-1^·min^-1^ at 500 rpm (Fig 2B). Similarly, the effect of aeration rate on reaction velocity was also analyzed (Fig 2C). The reaction velocity at 1.5 vvm was the highest (0.32 ± 0.02 g·L^-1^·min^-1^). Any further increases of agitation speed or aeration rate resulted in a decrease of reaction velocity, probably due to the cell damage caused by high stirring shear.

The effect of substrate concentration on the conversion ratio was determined on the basis of the optimal agitation speed and aeration rate (Fig 2D). The 100% of conversion was obtained at 20 g·L^−1^ and 30 g·L^−1^ l-phenylalanine within 2 h and 3 h, respectively. Although higher substrate concentrations increased the production, the conversion ratio dramatically decreased (i.e., 88.6% and 80.0% at 35 g·L^−1^ and 40 g·L^−1^ l-phenylalanine, respectively). Therefore, the optimal l-phenylalanine concentration was 30 g·L^−1^ for resting cell biotransformation in the 3-L bioreactor and the corresponding productivity was 10.0 g·L^-1^·h^-1^, which was 6 times that in the flask.

**Fig 3:**
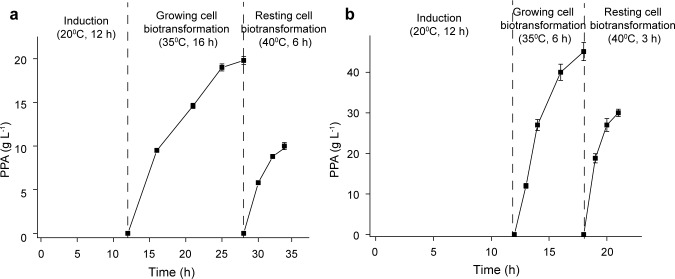
Time profiles of PPA production using growing and resting cell biotransformation in the flask (a) and 3-L bioreactor (b).

Fig 3 shows the time courses of two-step production of PPA involving growing and resting cells in the flask (Fig 3A) and 3-L bioreactor (Fig 3B), respectively. Under growing conditions in nutrient medium, two-stage temperature control was performed, and then the catalysts were collected and washed, followed by resting cell conditions in saline solution. The PPA production in the 3-L bioreactor (75.1 ± 2.5 g·L^−1^) was 2.5 times that in the flask (29.8 ± 2.1 g·L^−1^), due to good oxygen transfer which was favorable for cell growth and whole-cell biotransformation [25, 26]. The production of growing cell biotransformation was 2.0 and 1.5 times that of resting cell biotransformation in the flask and 3-L bioreactor, respectively, probably due to stable catalytic performance of the recombinant *E*. *coli* under growing conditions. Although the PPA production by the growing cell transformation is an attractive alternative, the resting cell biotransformation showed shorter reaction time, convenient product separation, and the capacity to be recycled several times without obvious decreases in activity, which are also important considerations for industrial PPA production.

Compared with one-step biotransformation used in the previous studies [9], the two-step biotransformation showed no decreased production during resting cell biotransformation, while an appreciable PPA production of growing cell biotransformation was obtained. As a result, the total PPA titer of the two-step biotransformation was 3.0 and 2.5 times that of the by one-step biotransformation in the flask and 3-L bioreactor, respectively. Therefore, the two-step biotransformation is a promising method for the industrial production of PPA.

Two-step biotransformation involving growing and resting cells was widely used in the production of many compounds by different strains. It is not convenient to perform genetic manipulation for these strains due to insufficient genetic information, which is unfavorable for the metabolic pathway engineering. Furthermore, these intracellular reactions limited the combination of substrate and enzymes and the release of product. Compared with the previous studies, our two-step biotransformation system showed certain advantages. For example, the well-characterized host *E*. *coli* was used and this made it convenient to perform pathway engineering and molecular engineering of l-AAD, and the substrate is more easily combined with the enzyme without any obstacle due to the membrane-bound nature of l-AAD.

These results indicate that high l-phenylalanine concentration is unfavorable for oxidative deamination in the 3-L bioreactor, which was consistent with a previous study [9]. This inhibition may be attributed to the relatively low dissolution of oxygen, biocatalyst deactivation, substrate inhibition, and product inhibition. Therefore, based on the initial reaction rate, a kinetic model was developed to determine the possible ways to overcome the disadvantages.

### Effect of dissolved oxygen, cell, substrate, and product concentration on initial rates

**Fig 4:**
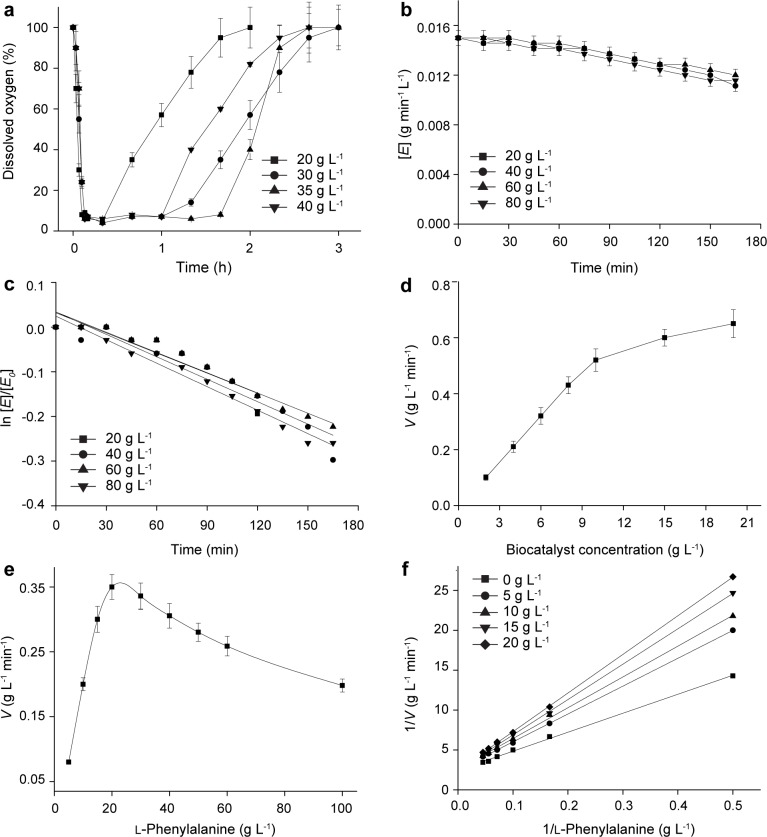
Kinetic analysis of cell deactivation, substrate and product inhibition of the resting cell biotransformation in the 3-L bioreactor. a: time profiles of dissolved oxygen with different l-phenylalanine concentrations. b: time profiles of cell deactivation at different l-phenylalanine concentrations. c: calculation of the deactivation constant. d: effect of biocatalyst concentration on the initial rate. e: effect of substrate concentration on the initial reaction rate. f: Lineweaver-Burk plotting with different PPA addition.

To evaluate the effect of dissolved oxygen, the time courses for dissolved oxygen with different l-phenylalanine concentrations were compared (Fig 4A). At the beginning of the reaction, the dissolved oxygen was decreased to below 10% within 10 min, indicating that the initial reaction velocity was high and a large amount of oxygen was required. When the reaction velocity was decreased with time, the dissolved oxygen was also increased and finally reached the saturation level (100%) when the reaction was stopped. However, the dissolved oxygen was recovered to its initial level at different times regardless of the l-phenylalanine concentration, indicating that the dissolved oxygen concentration was enough for resting cell biotransformation in the 3-L bioreactor.

Many factors, like toxic substrate [27], temperature [28], and pH [29], cause enzyme and whole-cell biocatalyst deactivation in the biotransformation. For resting cell biotransformation in the 3-L bioreactor, the whole-cell activity may decrease along with reaction time due to mechanical agitation and aerate. Therefore, effect of reaction time on cell deactivation was investigated under different l-phenylalanine concentrations. (Fig 4B). It shows that cell deactivation is a function of reaction time, while l-phenylalanine concentration has little influence on the cell activity. A possible relationship for the present deactivation of resting cell biocatalyst can be presented as Eq (1):

$$\left[E]=[E_{0}]•\text{exp}(\text{-}k_{\text{d}}•t\right)$$ (1)

where [*E*] is the cell activity (the amount of PPA produced by one mg recombinant *E*. *coli* per min; g·min^-1^·mg^-1^), [*E*_0_] is the initial cell activity (g·min^-1^·mg^-1^), *k*_d_ is the deactivation constant, and *t* is time (min). As shown in Fig 4C, when ln [*E*]/[*E*_*0*_] was plotted against *t* for each l-phenylalanine concentration, a series of linear relationship was obtained and the average *k*_d_ value was 1.61×10^−3^ ± 1.12×10^−4^ with the correlation coefficient factor R^2^ = 0.9912.The effect of the cell concentration on the initial rate is shown in Fig 4D. The initial rates of PPA formation were directly proportional to the resting cell concentration up to 10 g·L^-1^ cell biocatalyst, after which the relationship declined from the linearity. Eq (2) shows the linear relationship between initial rate and resting cell concentration, with the R^2^ for the linear fitting being 0.9878, respectively:

$$\frac{\text{d}[P]}{\text{d}t}\propto [E],(0\leq [E]\leq 10\text{g}/\text{L})$$ (2)

where [*P*] is the PPA concentration (g·L^-1^).

In the previous studies, we found that substrate inhibition exists in the l-AAD-based whole-cell biotransformation in the flask [9]. Therefore, the effect of l-phenylalanine concentration on the initial rate was also investigated (Fig 4E). The initial rate was almost proportional to the l-phenylalanine concentration when the concentration was below 30 g·L^−1^, after which the initial rate decreased along with the increasing l-phenylalanine concentration, indicating that the substrate inhibition existed during the resting cell biotransformation in the 3-L bioreactor. Therefore, the l-phenylalanine concentration *vs*. initial rate were well correlated with R^2^ = 0.9971 by linear fit and the substrate inhibition constant *K*_SI_ was 70.6 ± 2.5 g·L^−1^. The relationship between initial rate and l-phenylalanine concentration is provided in Eq (3):

$$\frac{\text{d}[P]}{\text{d}t}\propto \frac{\left[S\right]}{K_{\text{m}}+[S]•(1+\frac{\left[S\right]}{K_{\text{SI}}})}$$ (3)

where [*S*] is the l-phenylalanine concentration (g·L^-1^), *K*_m_ is the apparent Michaelis constant (g·L^-1^), and *K*_SI_ is the substrate inhibition constant (g·L^−1^). As shown in Fig 4F and Table 1, the apparent maximum reaction rate *V*_max_ values were unchanged, while the apparent Michaelis Sconstant *K*_m_ values increased along with the PPA concentration, demonstrating a competitive inhibition mode of PPA on the resting cell biotransformation. The PPA addition *vs*. apparent Michaelis constant *K*_m_ values were well correlated with R^2^ = 0.9762 by linear fit and the inhibition constant *K*_PI_ value was 22.3 ± 1.2 g·L^−1^. The relationship between initial rate and PPA concentration is provided in Eq (4):

$$\frac{\text{d}[P]}{\text{d}t}\propto \frac{\left[S\right]}{K_{\text{m}}•(1+\frac{\left[P\right]}{K_{\text{PI}}})+[S]}$$ (4)

where *K*_PI_ is the product inhibition constant (g·L^-1^).

**Table 1:** Comparison of *V*_*max*_ and *K*_*m*_ of the resting cell biotransformation with different PPA concentrations.

PPA concentration (g·L^−1^·min^−1^)	0	5	10	15	20
*K*_*m*_ (g·L^−1^)	9.83 ± 0.28	13.47 ± 0.42	15.02 ± 0.45	17.73±0.53	19.37 ± 0.58
*V*_*max*_ (g·L^−1^·min^−1^)	0.39 ± 0.01	0.40 ± 0.01	0.40 ± 0.01	0.41 ± 0.01	0.41 ± 0.01

### Development of model and experimental determination of overall rate constants

**Fig 5:**
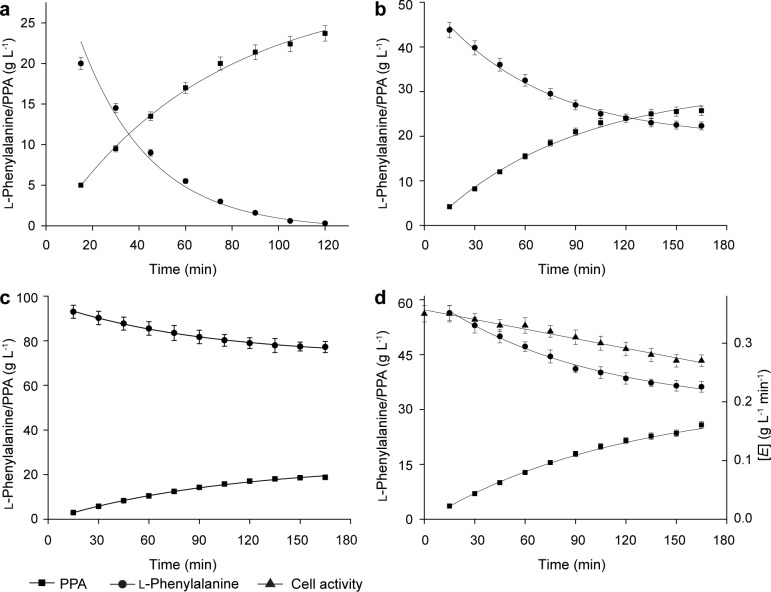
Batch biotransformation kinetics and model fitting to determine the overall rate constant. a: batch biotransformation at 24 g·L^-1^ l-phenylalanine with 7 g·L^-1^ cell. b: time course of batch biotransformation at 48 g·L^-1^ l-phenylalanine with 7 g·L^-1^ cell. c: batch biotransformation at 96 g·L^-1^ l-phenylalanine with 5 g·L^-1^ cell. d: evaluation of the model at 60 g·L^-1^ l-phenylalanine with 5 g·L^-1^ cell.

We then developed a kinetic model for the PPA production by whole cell biotransformation. Rate equations for PPA production, l-phenylalanine consumption, and cell deactivation are shown as Eqs (5), (6) and (7). Numerical integration with a time increment (Δ*t*) of 0.01 h was used to construct the simulation profiles. Eqs (8), (9) and (10) were used to determine the l-phenylalanine concentration, PPA production, and cell activity from the assigned time increment and rate equations:

$$\frac{\text{d}[P]}{\text{d}t}|\begin{array}{c} \text{i} \end{array}=\frac{V_{\text{p}}•[S_{\text{i}}]•[E_{\text{i}}]}{K_{\text{m}}•(1+\frac{\left[P_{\text{i}}\right]}{K_{\text{PI}}})+[S_{\text{i}}]•(1+\frac{\left[S_{\text{i}}\right]}{K_{\text{SI}}})}$$ (5)

$$\frac{\text{d}[S]}{\text{d}t}|\begin{array}{c} \text{i} \end{array}=-\frac{\text{d}[P]}{\text{d}t}|\begin{array}{c} \text{i} \end{array}$$ (6)

$$\frac{\text{d}[E]}{\text{d}t}|\begin{array}{c} \text{i} \end{array}=-k_{\text{d}}•[E_{\text{i}}]$$ (7)

$$[P_{\text{i}}]=[P_{\text{i-}1}]+\frac{\text{d}[P]}{\text{d}t}|\begin{array}{c} \text{i-}1 \end{array}•\Delta t$$ (8)

$$[S_{\text{i}}]=[S_{\text{i-}1}]-\frac{\text{d}[P]}{\text{d}t}|\begin{array}{c} \text{i-}1 \end{array}•\Delta t$$ (9)

$$[E_{\text{i}}]=[E_{\text{i-}1}]+\frac{\text{d}[P]}{\text{d}t}|\begin{array}{c} \text{i-}1 \end{array}•\Delta t$$ (10)

where *V*_P_ is the overall rate constant for PPA formation (the amount of PPA produced per min, g·L^-1^·min^-1^), i is the iteration loop identifier in numerical integration, and Δ*t* is step size constant (0.01 h). Three batches of biotransformation studies were used to calculate the overall rate constant (Fig 5A, 5B and 5C). The overall rate constant was *V*_p_ = 0.35 ± 0.01 g·L^-1^·min^-1^ based on the model with these parameters providing the ‘best fit’ between the experimental and predicted values of l-phenylalanine and PPA. The R^2^ corresponding to each fitting was 0.9965 for 24 g·L^-1^ l-phenylalanine with 7 g·L^-1^ cell, 0.9962 for 48 g·L^-1^ l-phenylalanine with 7 g·L^-1^ cell, and 0.9987 for 96 g·L^-1^ l-phenylalanine with 5 g·L^-1^ cell.

For the evaluation of the kinetic model of the reaction system developed here, the time course of l-phenylalanine consumption, cell deactivation, and PPA formation with an initial l-phenylalanine 60 g·L^-1^ and cell 5 g·L^-1^ were compared with a simulation of this conversion experiment by using the model. As shown in Fig 5D, the simulation profile was in close agreement with the experimental data with R^2^ = 0.9939. Therefore, it confirmed that the kinetic model was accurately predicted for the experimental results within this range of initial conditions. This model provides a more complete understanding of the kinetics of PPA production, as well as a foundation for optimization of substrate concentration in future batch, fed-batch or continuous processes.

The resting cell biotransformation showed significant substrate and product inhibition. Similar phenomena were observed for the other amino acid deaminases from *P*. *mirabilis* [30] and amino acid oxidases from *Rhodococcus opacus* [31] and *Pseudomonas putida* [32]. The removal of substrate inhibition using the protein engineering techniques has been successfully used in many enzymes such as l-lactate dehydrogenase from *Bacillus stearothermophilus* [33], β-N-acetyl-D-hexosaminidases from *Ostrinia furnacalis* [34], and betaine aldehyde dehydrogenase from *Staphylococcus aureus* [35]. Due to the low similarity (19%) of l-AAD to other l-amino amino oxidases, it is difficult to develop a homology model to eliminate the substrate and product inhibition by directional protein engineering. Therefore, the current study proposed a simple kinetic model of PPA biotransformation by *E*. *coli* resting cells based on kinetic analysis of the effects of substrate and product inhibition on the initial reaction rate. This model provides a more complete understanding of the kinetics of PPA production as well as the foundation for optimization of substrate concentrations in batch, fed-batch, or continuous processes.

## Conclusions

We produced PPA with a high production titer as well as high productivity from l-phenylalanine by metabolically engineered *E*. *coli* in a new two-step biotransformation system. The reaction conditions were then optimized in the flask and 3-L bioreactor, respectively. The two-stage temperature control was performed to improve the production and cell growth in growing cell biotransformation. Future work will focus on elimination of substrate and product inhibition by protein engineering techniques and based on the kinetic model of PPA biotransformation.
